# Effectiveness of Non-Medical Treatment on Survival, Perioperative, and Clinical Outcomes in Canine Cushing’s Syndrome: A Systematic Review and Network Meta-Analysis

**DOI:** 10.3390/vetsci13080771

**Published:** 2026-08-01

**Authors:** Sophia Shanlly, Jordan Slessor, Wenting Yan, Eva D. C. Rosaasen-Uwiera, Jessica J. D. Thorlakson, Heather L. Bruce, Richard R. E. Uwiera

**Affiliations:** 1 Department of Agricultural, Food & Nutritional Science, University of Alberta, Edmonton, AB T6G 2R3, Canada; sshanlly@ualberta.ca; 2 Department of Mathematical & Statistical Sciences, University of Alberta, Edmonton, AB T6G 2R3, Canada; jaslesso@ualberta.ca; 3 Faculty of Nursing, University of Alberta, Edmonton, AB T6G 2R3, Canada; wyan4@ualberta.ca; 4 Department of Psychology, University of Alberta, Edmonton, AB T6G 2R3, Canada; euwiera@ualberta.ca; 5 Cameron Science and Engineering Library, University of Alberta, Edmonton, AB T6G 2R3, Canada; jthorlak@ualberta.ca; 6 Department of Animal Science and Aquaculture, Dalhousie University, Truro, NS B2N 5E3, Canada; heather.bruce@dal.ca

**Keywords:** canine, Cushing’s syndrome, surgical treatment, adrenalectomy, radiotherapy

## Abstract

Cushing’s syndrome is a common endocrine disorder in dogs managed with both medical and non-medical treatments; however, the effects of non-medical interventions on survival, perioperative, and clinical outcomes remain uncertain. A comprehensive search was conducted across MEDLINE, Embase, Web of Science, Academic Search Complete, and the Cochrane Library databases. Five studies (*n* = 248 dogs) met the inclusion criteria. Three studies evaluated laparoscopic and open adrenalectomy in dogs with adrenal-dependent hypercortisolism, whereas two studies evaluated radiotherapy in dogs with pituitary-dependent hypercortisolism. Meta-analysis demonstrated no significant differences in the survival rates between laparoscopic and open adrenalectomy at 12, 24, or 36 months. Fractionated radiotherapy improved survival rates at 12 months, while both fractionated and stereotactic radiotherapy improved survival rates at 24 months. This benefit, however, was not maintained at 36 months. Radiotherapy was also associated with improved neurologic outcomes (OR = 109.17, 95% CI 3.99–2985.49) and longer survival time (MD = 624.00 days, 95% CI 227.93–1020.07). Among dogs undergoing adrenalectomies; laparoscopic adrenalectomy was associated with shorter surgical times (MD = −28.38 min, 95% CI −41.39 to −15.38) and hospitalization (MD = −0.66 days, 95% CI −1.00 to −0.32) compared to open adrenalectomy.

## Simple Summary

Cushing’s syndrome is a common endocrine disorder in dogs that is associated with long-term elevated glucocorticoid activity and cortisol overproduction. Although medical treatment is commonly used, non-medical treatments such as surgery and radiotherapy are important alternatives in selected cases. The effects of non-medical treatments on survival, perioperative, and other clinical outcomes, however, have not been fully evaluated. This systematic review and meta-analysis evaluated the available evidence for treatment outcomes of adrenalectomy in dogs with adrenal-dependent hypercortisolism and radiotherapy in dogs with pituitary-dependent hypercortisolism. Five studies, with 248 dogs, met the inclusion criteria. No significant differences in survival rates were observed between laparoscopic and open adrenalectomy at 12, 24, or 36 months. However, laparoscopic adrenalectomy was associated with shorter surgical times and a reduced period of hospitalization. Radiotherapy for pituitary tumors improved short- to medium-term survival rates compared with no treatment, although this benefit was not sustained at longer follow-up intervals. Additionally, radiotherapy was associated with improved neurological outcomes and increased overall survival time. These findings support the possible use of non-medical interventions in appropriately selected dogs with Cushing’s syndrome. Noteworthy, the evidence is limited to a small number of retrospective studies, and further prospective research should be considered to support these findings.

## 1. Introduction

Cushing’s syndrome (CS) is a common endocrine disorder in dogs, characterized by chronic excess glucocorticoid activity. In accordance with the second cycle of “Agreeing Language in Veterinary Endocrinology” (ALIVE), Cushing’s syndrome is classified as either iatrogenic or naturally occurring and is associated with elevations in serum steroid hormone levels [1]. Naturally occurring CS develops from dysregulated cortisol secretion, and this results from either abnormalities of the hypothalamic–pituitary–adrenal (HPA) axis or autonomous cortisol secretion by the adrenal cortex [2]. It is estimated that naturally occurring CS affects approximately 1–2 dogs per 1000 [2]. In contrast to naturally occurring CS, iatrogenic CS results from the prolonged administration of exogenous glucocorticoids and is less common than naturally occurring CS in dogs [1].

Naturally occurring CS is further classified into adrenocorticotropic hormone (ACTH)-dependent or ACTH-independent forms of the disorder. Approximately 80–85% of cases are ACTH-dependent and are most commonly caused by pituitary-dependent hypercortisolism (PDH). PDH is often associated with excessive ACTH secretion from a pituitary adenoma. Less commonly, however, PDH is linked to pituitary gland hyperplasia and increased ACTH secretion, and this stimulates bilateral adrenal hyperplasia leading to sustained cortisol production [2,3,4,5]. Clinical signs of PDH can include polyuria, polydipsia, polyphagia, abdominal distension due to fat redistribution, and hepatomegaly [6]. In addition, dermatologic abnormalities such as alopecia, thinning of the skin, and impaired wound healing can also occur in dogs with PDH [6]. While medical therapy remains the primary treatment for most dogs with PDH, hypophysectomy or radiotherapy can also be considered. These treatments are particularly useful in dogs with large pituitary tumors, neurological abnormalities, or other clinical manifestations of the disease that remain in refractory to medical management [6].

Approximately 15–20% of naturally occurring CS cases are ACTH-independent forms and are most commonly associated with adrenal-dependent hypercortisolism (ADH) [1]. ADH most often results from uncontrolled cortisol secretion from adrenal gland cortical adenomas or carcinomas [4,5]. Excess cortisol production often arises from a single adrenal gland, and this can result in atrophy of the contralateral adrenal gland due to the suppression of pituitary ACTH secretion [5]. Clinical signs commonly include polyuria, polydipsia, polyphagia, abdominal enlargement, and in some incidences, an intra-abdominal adrenal mass can be palpated [5,6]. Adrenalectomy is considered the treatment of choice for dogs with adrenal tumors, and this procedure can be curative with the complete excision of the mass. The dog’s recovery and remission of disease, however, depends on tumor type, local tissue invasion, vascular involvement, and distant tumor cell metastasis; with adrenal carcinomas being generally associated with less favorable long-term outcomes [6]. Other rarer causes of naturally occurring CS have been documented, and these include ectopic sources of ACTH secretion and the aberrant ACTH receptor expression on adrenal glands (including food-dependent hypercortisolism) [1,6].

Management of naturally occurring CS in dogs can be separated into medical and non-medical treatments. Medical therapy is most common and targets adrenal steroidogenesis or pituitary regulation. Drugs such as trilostane (a competitive inhibitor of 3β-hydroxysteroid dehydrogenase), mitotane (an adrenocorticolytic agent), ketoconazole (an inhibitor of steroid synthesis), cabergoline (a dopamine agonist), selegiline (a monoamine oxidase-B inhibitor), and aminoglutethimide (a steroidogenesis inhibitor) are employed to reduce cortisol production or modulate ACTH secretion [7,8,9,10,11,12].

Non-medical interventions are used less frequently but remain important for treatment in selected cases. These interventions are directed to reduce (shrink tumor size) or remove (tumor excision) the underlying source of excessive cortisol production. Adrenalectomy involves surgical removal of the affected adrenal gland and is primarily performed in dogs with localized adrenal tumors [13]. Hypophysectomy involves surgical excision of the pituitary gland, and may be considered in selected dogs with PDH and identifiable pituitary masses [14]. Radiotherapy uses ionizing radiation, typically delivered through high-energy photon beams, to target pituitary tumors [15]. Radiotherapy is primarily used to reduce tumor size and progression, and can improve neurologic signs associated with pituitary macroadenomas and other pituitary masses [15].

At present, no systematic review and meta-analysis have evaluated the effects of non-medical interventions on survival, perioperative, and clinical outcomes within dogs with CS. Therefore, the objective of this systematic review and meta-analysis was to evaluate the available evidence of adrenalectomy and radiotherapeutic interventions for CS in dogs. Notably, outcomes of adrenalectomy were determined only from studies involving ADH dogs, whereas outcomes of radiotherapy were only determined from studies involving PDH dogs. The findings of this study aim to support future evidence-based treatments of different subtypes of CS and to identify areas that may require further research.

## 2. Materials and Methods

### 2.1. Search Strategy

This systematic review and meta-analysis followed the Preferred Reporting Items for Systematic Reviews and Meta-Analyses (PRISMA) 2020 guidelines and the Cochrane Review Methods. Prior to data extraction and analysis, the protocol was registered with the Open Science Framework on 5 October 2025 (https://doi.org/10.17605/OSF.IO/2P7F9). A comprehensive search was performed in MEDLINE (1946–present via Ovid), Embase (1974–present via Ovid), Web of Science–All Databases (Clarivate Analytics), Academic Search Complete (EBSCO), and the Cochrane Library (Wiley Online Library) from 1 September 2025 to 3 October 2025 to identify all relevant studies evaluating surgical and radiotherapeutic interventions for CS in dogs. The following search terms were applied: (exp Dogs/OR canine* OR dog OR dogs OR doggy OR puppy OR puppies OR mongrel* OR hound OR hounds OR terrier* OR spaniel* OR retriever* OR mastiff* OR pinscher* OR collie* OR poodle* OR dachshund* OR corgi* OR shepherd* OR sheepdog* OR beagle* OR coonhound* OR bloodhound* OR borzoi* OR english foxhound* OR greyhound* OR harrier* OR irish wolfhound* OR otterhound* OR rhodesian ridgeback* OR scottish deerhound* OR pooch* OR mutt OR mutts OR bitch*) AND (exp Cushing Syndrome/OR exp Pituitary ACTH Hypersecretion/OR exp ACTH-Secreting Pituitary Adenoma/OR exp Adrenal Gland Neoplasms/OR exp Adrenocorticotropic Hormone/OR exp Adrenocortical Hyperfunction/OR exp Hydrocortisone/OR cushing* OR adrenocorticotropic hormone* OR ACTH* OR ((corticotrop* OR pituitary OR adrenal) ADJ2 (adenoma* OR tumo?r*)) OR hyperadrenocortic* OR hypercortisol* OR hydrocortisone*) AND (exp General Surgery/OR Hypophysectomy/OR exp Adrenalectomy/OR exp Radiotherapy/OR surgical* OR surger* OR transsphenoidal* OR hypophysectom* OR adrenalectom* OR neurosurger* OR neurosurgical* OR radiotherap* OR radiotherapeutic* OR radiosurger* OR radiat* OR cyberknife* OR gamma knife* OR resection* OR excision*). Exploded Medical Subject Headings (MeSH) were used where appropriate, and all identified keywords and index terms were adapted for each individual database to ensure a comprehensive search strategy. No restrictions were applied regarding language, publication date, or publication status, allowing the inclusion of all potentially relevant studies. Reference lists of included articles were manually screened, and Google Scholar was searched to capture any additional eligible publications not indexed in the selected databases. Detailed search strategies for each database are presented in Supplementary Materials, Tables S1–S5. All search results were imported into Covidence, and duplicate records were identified and removed prior to the review process.

### 2.2. Eligibility Criteria

The primary literature included in this systematic review and meta-analysis met predefined inclusion and exclusion criteria. Eligible studies involved dogs diagnosed with all CS of any type, regardless of the stage of disease or comorbidities, and reported outcomes for at least one non-medical treatment; including adrenalectomy, hypophysectomy, or radiotherapy. Each study was required to include a comparator group, with comparisons permitted between two or more non-medical treatments, between a non-medical treatment and medical treatment, or between a non-medical treatment and no treatment (NT), and to report outcome measures related to survival time and/or survival rate. Only randomized controlled trials (RCTs) and cohort studies (prospective or retrospective) were considered for the analysis. Studies that were in vitro, case reports, or lacked in a comparator group were also excluded. Additionally, reviews, editorials, letters, and non-peer-reviewed publications were not considered eligible for this study.

### 2.3. Data Extraction

Screening and study selection were completed in Covidence (Veritas Health Innovation, Melbourne, Australia). Two reviewers (S.S. and W.Y.) independently screened the titles, abstracts, and full-text articles based on predetermined eligibility criteria. Any disagreements and final inclusion decisions were resolved by two additional reviewers (R.R.E.U. and E.D.C.R.U.) to ensure consensus. Data extracted from each study included study identification, methodological design, population characteristics, details of interventions and comparators, outcome measures, and numerical results. Outcome data reported in non-standard units were converted to standardized formats, and standard deviation (SD) was calculated from the standard error of the mean (SEM) when SEM was the only value provided.

### 2.4. Quality Assessment

Methodological Quality of included studies was evaluated using the Cochrane Risk of Bias in Non-Randomized Studies of Interventions (ROBINS-I) tool. Two reviewers (S.S. and J.S.) independently assessed each study for potential sources of bias across seven domains, including confounding, participant selection, intervention classification, deviations from intended interventions, incomplete outcome data, outcome measurement, and selective reporting of results. Any disagreements were addressed through discussion until consensus was achieved. Detailed risk of bias for included studies is presented in Supplementary Materials, Tables S6–S10.

### 2.5. Statistical Analysis

Statistical analyses were conducted using both pairwise and network meta-analysis. Pairwise meta-analyses were performed using Review Manager (RevMan) version 5.4.1 (The Cochrane Collaboration, Copenhagen, Denmark). Network meta-analyses were conducted using the *netmeta* package in R version 4.4.1 (R Foundation for Statistical Computing, Vienna, Austria). Pairwise meta-analysis was used for direct comparisons between laparoscopic adrenalectomy (LA) and open adrenalectomy (OA) with pooled estimates calculated using the inverse variance method within a random-effects model to account for heterogeneity across studies. Dichotomous outcomes were expressed as odds ratios (OR) with 95% confidence intervals (CI), including survival rates at predefined follow-up intervals and perioperative survival. Continuous outcomes were summarized using mean differences (MD) with corresponding 95% CI, including surgical duration (minutes), length of hospitalization (days), and survival time (days). For studies reporting continuous outcomes as medians with 95% confidence intervals, the sample mean was estimated to be equal to the median, and the SD was estimated from the confidence limits and sample size [16,17]. These estimated means and standard deviations were subsequently used in the quantitative synthesis of continuous outcomes. Between-study heterogeneity was assessed using the I^2^ statistic and the Chi-squared test, with an I^2^ value greater than 50% indicating moderate to substantial heterogeneity. Statistical significance was defined as a two-sided *p*-value < 0.05. Network meta-analysis was used for both direct and indirect comparisons across stereotactic Radiotherapy (SRT), fractionated radiotherapy (FRT), and NT. The network meta-analysis allowed for the simultaneous evaluation of both direct (studies directly comparing the same interventions) and indirect (studies comparing interventions through a common comparator) data across various treatment comparisons. This provided a more comprehensive assessment of relative treatment effects than a conventional pairwise meta-analysis [18]. In this study, several assumptions, namely transitivity and consistency, were considered while assessing the data. Transitivity included network studies that were similar in both clinical and methodological characteristics to allow for comparisons of indirect data [18,19]. Consistency included both direct and indirect data to provide comparable estimates of treatment effects [18,19]. Although these assumptions were considered, the network meta-analysis consisted of only two retrospective studies for radiotherapies and as such statistical assessment of these assumptions was not possible. Consequently, the network meta-analysis should be interpreted cautiously, as the validity and precision of the indirect estimates may be limited. Finally, dichotomous outcomes were summarized as ORs and continuous outcomes as MDs, each with corresponding 95% CI. Publication bias was not evaluated because fewer than 10 studies were included in the meta-analysis [20]. Moreover, funnel plots and statistical tests for publication bias are considered unreliable when low numbers of studies are available [20]. As such, the assessment of publication bias had limited value for this study.

## 3. Results

### 3.1. Study Selection

**Figure 1:**
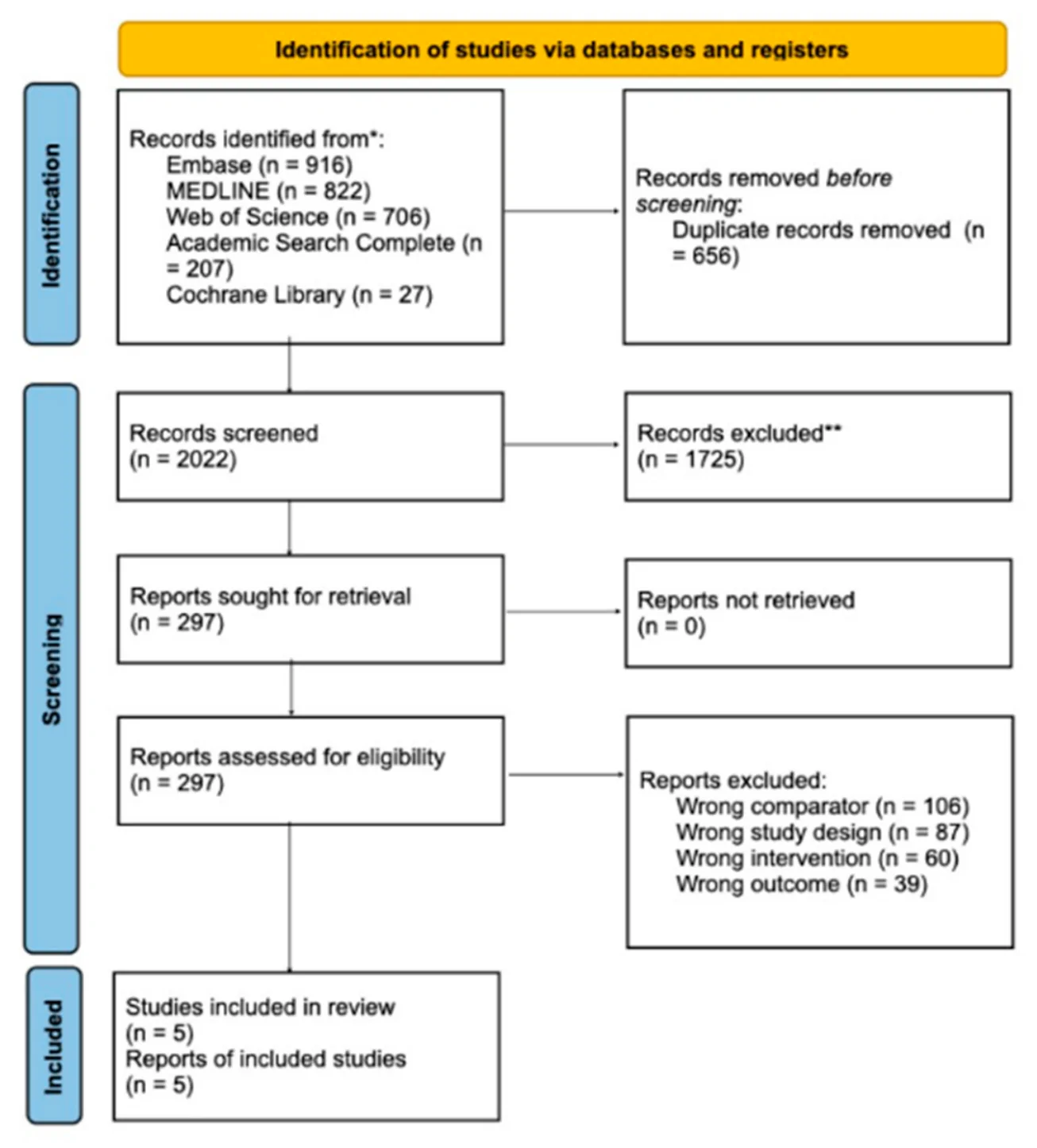
PRISMA 2020 flow diagram of the study selection process. * Records identified from electronic databases. ** Records excluded during title/abstract screening and full-text assessment according to the predefined eligibility criteria.

The database search identified 2678 records from five electronic databases: Embase (1974–present via Ovid; *n* = 916), MEDLINE (1946–present via Ovid; *n* = 822), Web of Science—All Databases (Clarivate Analytics; *n* = 706), the Cochrane Library (Wiley Online Library; *n* = 27), and Academic Search Complete (EBSCO; *n* = 207). After removal of 656 duplicate records, 2022 records were screened through title and abstract. Of these, 297 records were retrieved and assessed for eligibility through full-text review. Following full-text review, 292 records were excluded for the following reasons: ineligible comparators, ineligible study design, ineligible interventions, or ineligible outcomes. Five studies met the inclusion criteria and were included in the systematic review, and all five studies were included in the meta-analysis. The study selection process is presented in Figure 1.

### 3.2. Study Characteristics

Five studies met the inclusion criteria and included 248 dogs with naturally occurring CS. The study characteristics are summarized in Table 1. Eligible articles evaluated at least one non-medical intervention, LA, OA, SRT, or FRT, and reported outcomes. Three studies supported direct comparisons between LA and OA for adrenal tumor management, while two studies evaluated radiotherapy for pituitary masses, including comparisons between radiotherapeutic interventions and untreated control groups. All included studies were retrospective cohort studies, with variation in surgical treatment, radiotherapeutic intervention, and clinical management. Most subjects were middle-aged to older dogs of multiple breeds, with reported mean or median ages generally ranging from 8 to 11 years. Outcomes were reported as survival rates at predefined follow-up intervals (12, 24, and 36 months; %), median survival time (days), perioperative survival (%), surgical duration (minutes), length of hospitalization (days), and neurological outcomes (improvement or no improvement).

### 3.3. Risk of Bias

**Figure 2:**
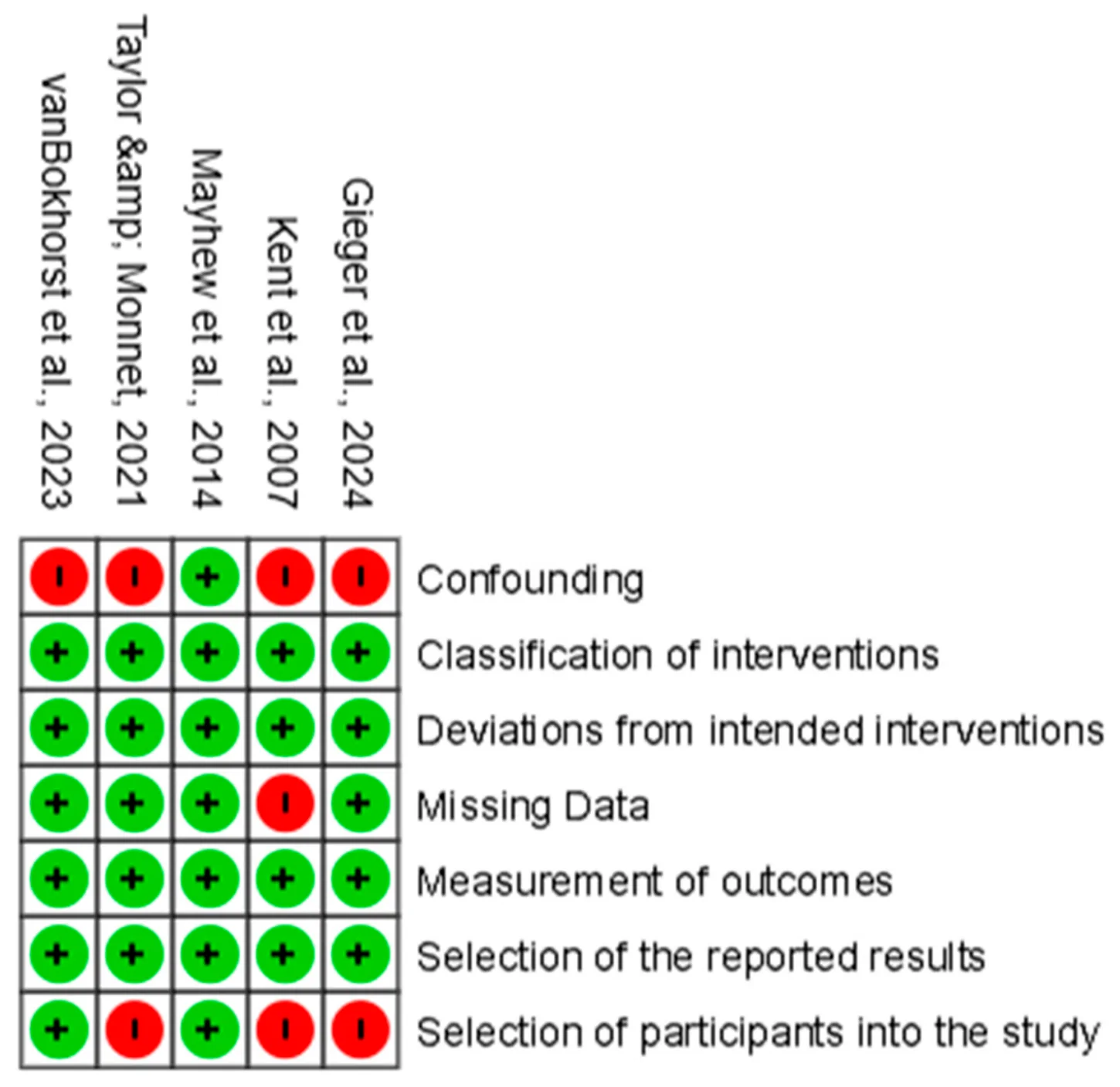
Cochrane risk of bias summary. Green circles indicate low risk of bias and red circles indicate high risk of bias across eight domains [20,21,22,23,24,25].

The risk of bias for the included studies was assessed using the Cochrane ROBINS-I tool, with results summarized in Figure 2. Most domains were rated as low risk of bias across the studies, including classification of interventions, deviations from intended interventions, measurement of outcomes, and selection of the reported results. Missing data was generally considered low risk, with only one study showing higher risk. Conversely, confounding bias was the primary limitation of the study, with four of the five studies rated as high risk. This reflected the non-randomized design of the studies and limited control of key clinical variables. Notably, only one study was assessed as low risk in this domain. Additionally, selection of participants into the study showed some variability, with three studies rated as high risk and two as low risk—indicating a potential bias related to participant inclusion. Although most domains demonstrated a low risk of bias; confounding, participant selection and missing data represent the main sources of potential bias in the included studies.

### 3.4. Adrenalectomy Survival Outcomes

**Figure 3:**
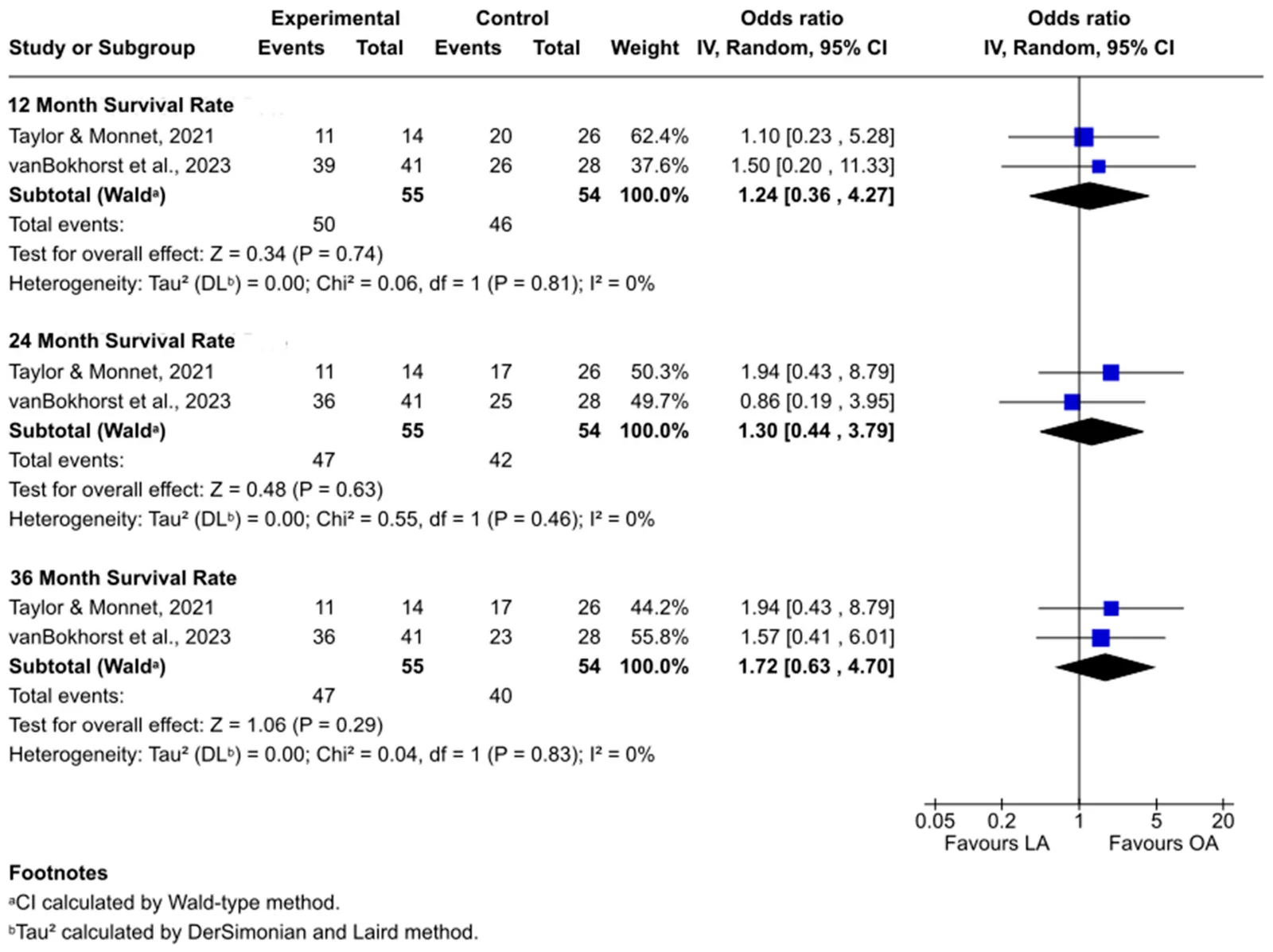
Forest plot comparing 12-, 24-, and 36-month survival rates in dogs undergoing LA versus OA across included studies [24,25].

**Figure 4:**
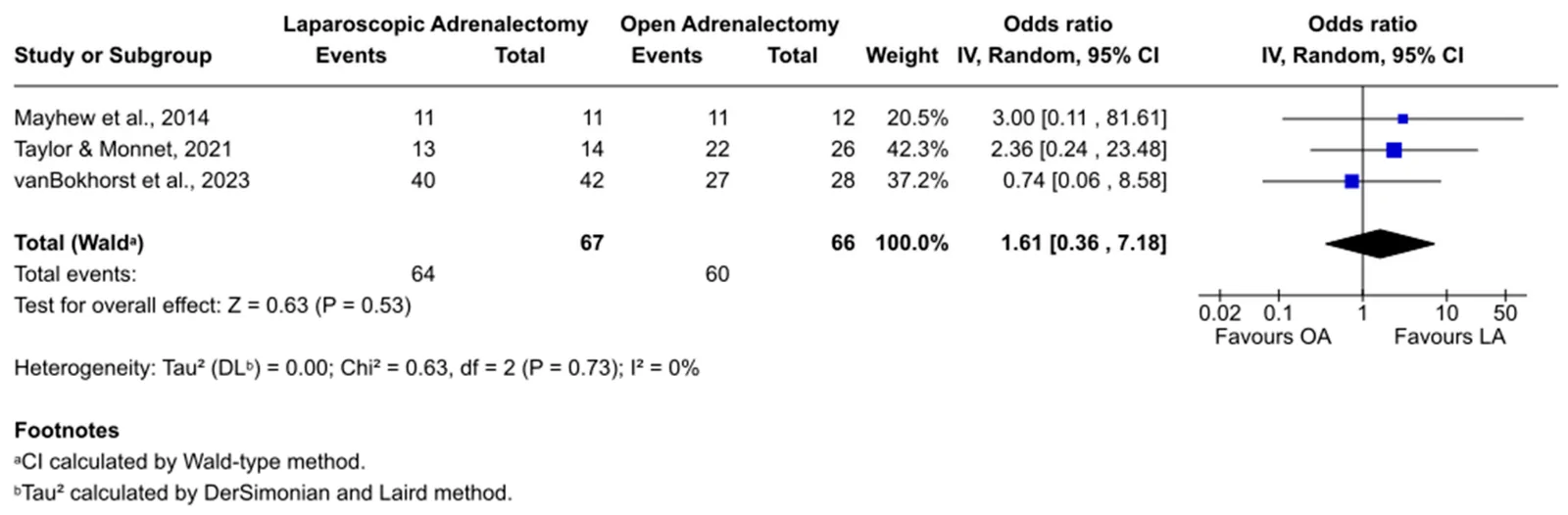
Forest plot comparing perioperative survival rates in dogs undergoing LA versus OA across included studies [23,24,25].

The meta-analysis demonstrated no statistically significant differences in survival rates between LA and OA at any evaluated time point. Survival rates at 12, 24, and 36 months are presented in Figure 3. At 12, 24 and 36 months the pooled OR was 1.24 (95% CI 0.36–4.27; *p* = 0.74), 1.30 (95% CI 0.44–3.79; *p* = 0.63) and 1.72 (95% CI 0.63–4.70; *p* = 0.29) respectively. No heterogeneity was observed across studies at any time point (12 months: I^2^ = 0%, *p* = 0.81; 24 months: I^2^ = 0%, *p* = 0.46; 36 months: I^2^ = 0%, *p* = 0.83). Across all time points, the wide CIs indicate limited precision, likely reflecting the small sample size of the studies. Perioperative survival outcomes are shown in Figure 4. No statistically significant difference was observed between LA and OA (OR = 1.61, 95% CI 0.36–7.18; *p* = 0.53), with no heterogeneity across studies (I^2^ = 0%, *p* = 0.73).

### 3.5. Adrenalectomy Perioperative Outcomes

**Figure 5:**
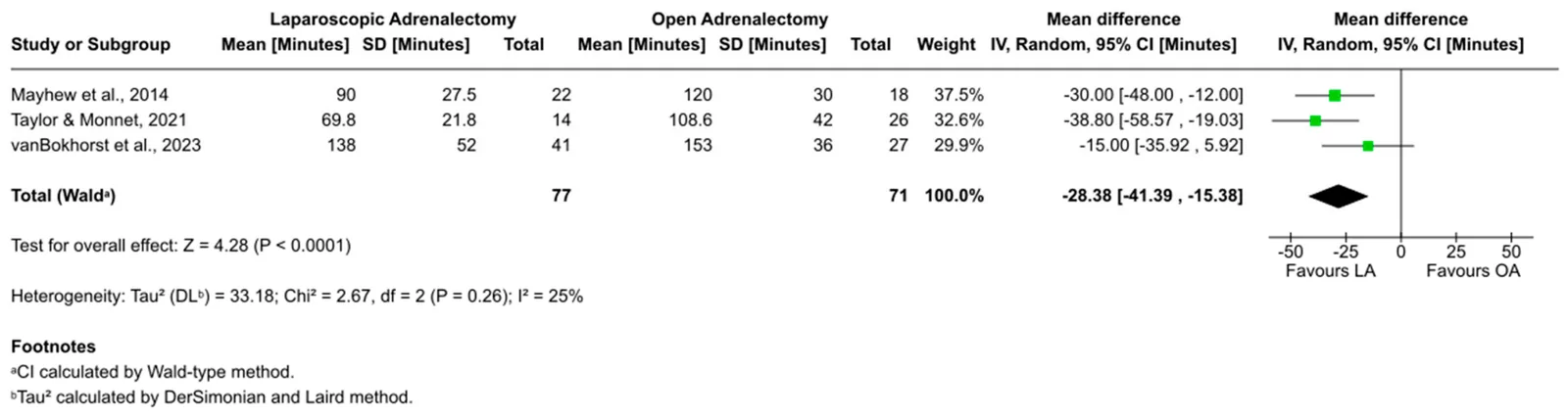
Forest plot comparing surgical time in dogs undergoing LA versus OA across included studies [23,24,25].

**Figure 6:**
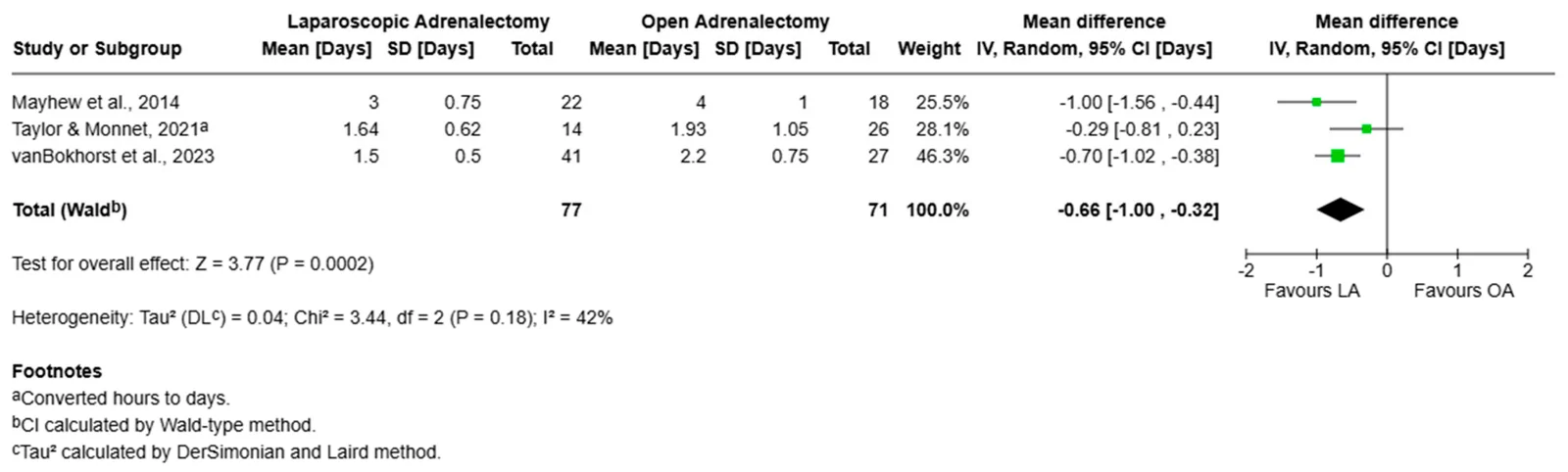
Forest plot comparing length of hospitalization in dogs undergoing LA versus OA across included studies [23,24,25].

The meta-analysis demonstrated statistically significant differences in perioperative outcomes. Perioperative outcomes are presented in Figure 5 and Figure 6. As compared to OA, LA was associated with a statistically significant reduction in surgical time (MD = −28.38 min, 95% CI −41.39 to −15.38; *p* < 0.0001; I^2^ = 25%, *p* = 0.26) and a shorter length of hospitalization (MD = −0.66 days, 95% CI −1.00 to −0.32; *p* = 0.0002; I^2^ = 42%, *p* = 0.18).

### 3.6. Radiotherapy Survival Outcomes

**Figure 7:**
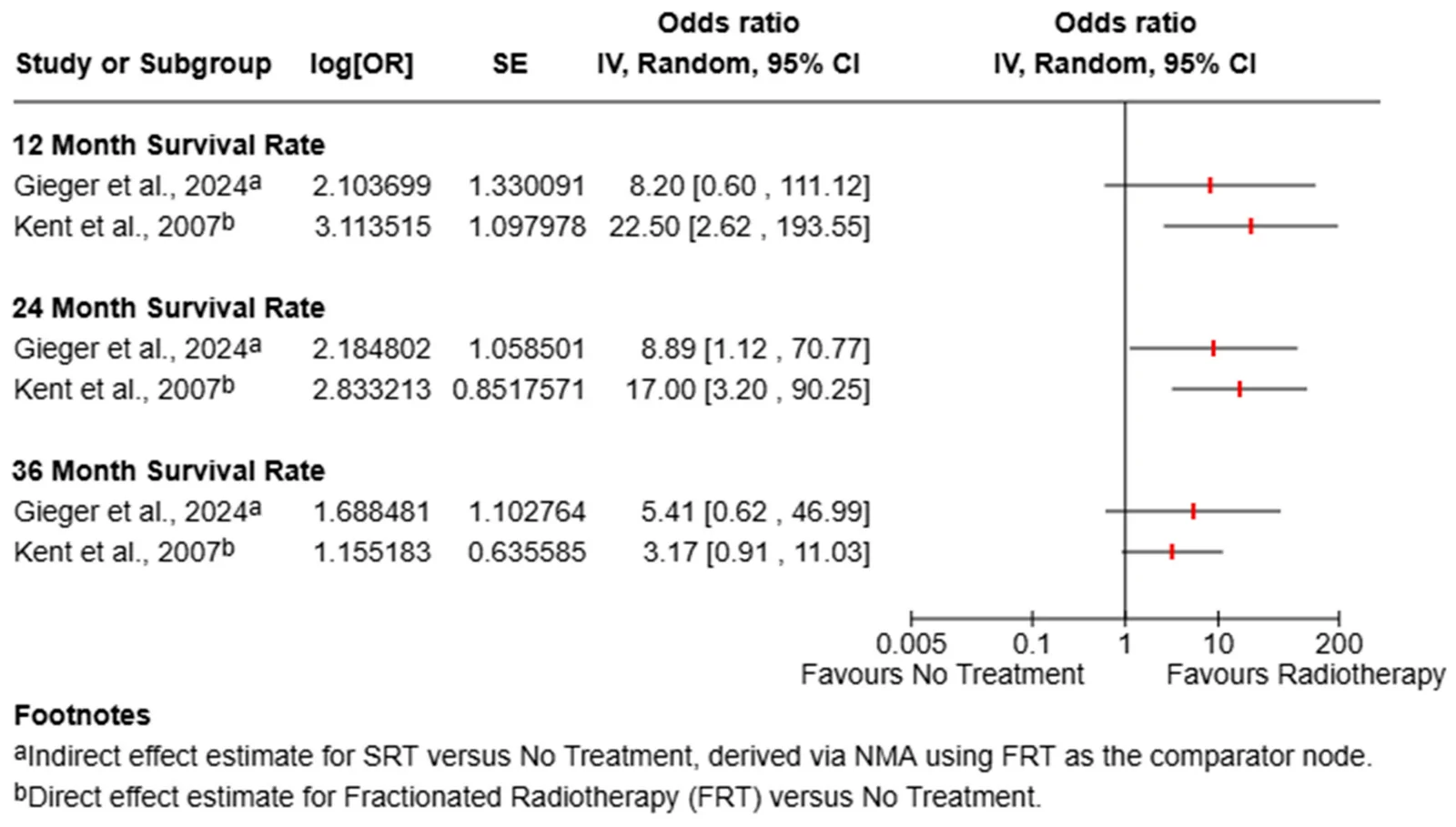
Forest plot comparing 12-, 24-, and 36-month survival rates in dogs treated with FRT and SRT versus NT across included studies [21,22].

The network meta-analysis demonstrated time-dependent effects of radiotherapy on survival outcomes. Survival rates at 12, 24, and 36 months are presented in Figure 7. At 12 months, FRT was associated with a statistically significant improvement in survival compared with NT (OR = 22.50, 95% CI 2.62–193.55; *p* = 0.0046). In contrast, SRT was not associated with a statistically significant improvement in survival relative to NT (OR = 8.20, 95% CI 0.60–111.12; *p* = 0.1137). At 24 months, both FRT and SRT demonstrated statistically significant survival benefits relative to NT (FRT: OR = 17.00, 95% CI 3.20–90.25; *p* = 0.0009; SRT: OR = 8.89, 95% CI 1.12–70.77; *p* = 0.0390). At 36 months, however, neither radiotherapeutic interventions provided a statistically significant survival advantage relative to NT (FRT: OR = 3.17, 95% CI 0.91–11.03; *p* = 0.0691; SRT: OR = 5.41, 95% CI 0.62–46.99; *p* = 0.1257).

### 3.7. Radiotherapy Clinical Outcomes

**Figure 8:**
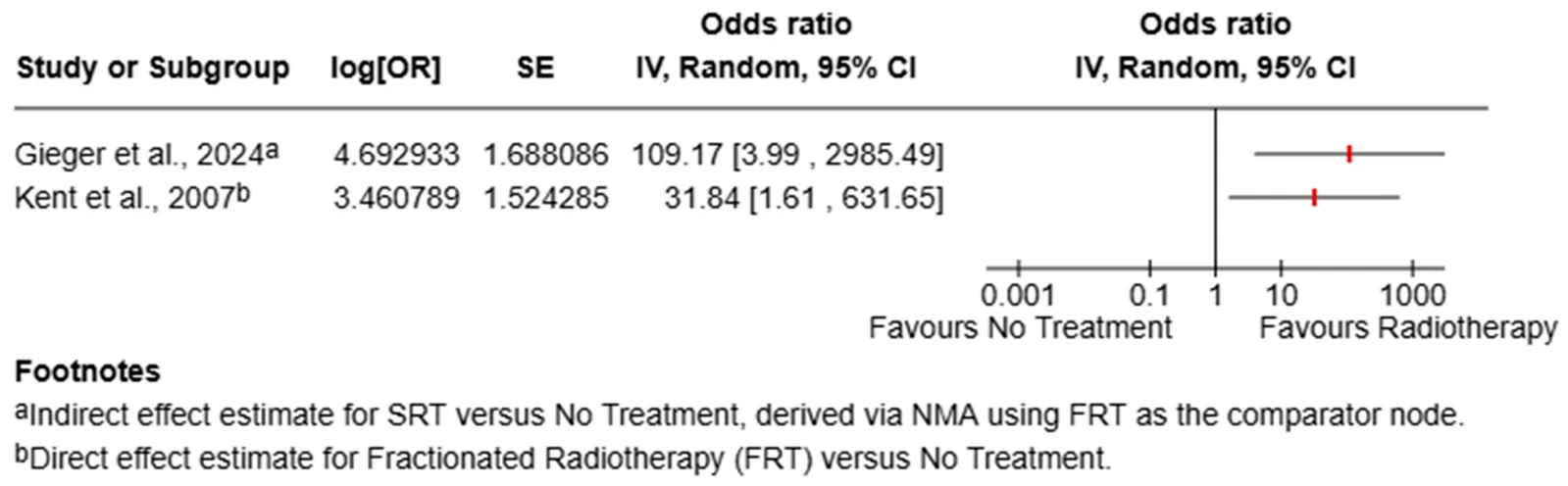
Forest plot comparing neurological improvements in dogs treated with FRT and SRT versus NT across included studies [21,22].

**Figure 9:**
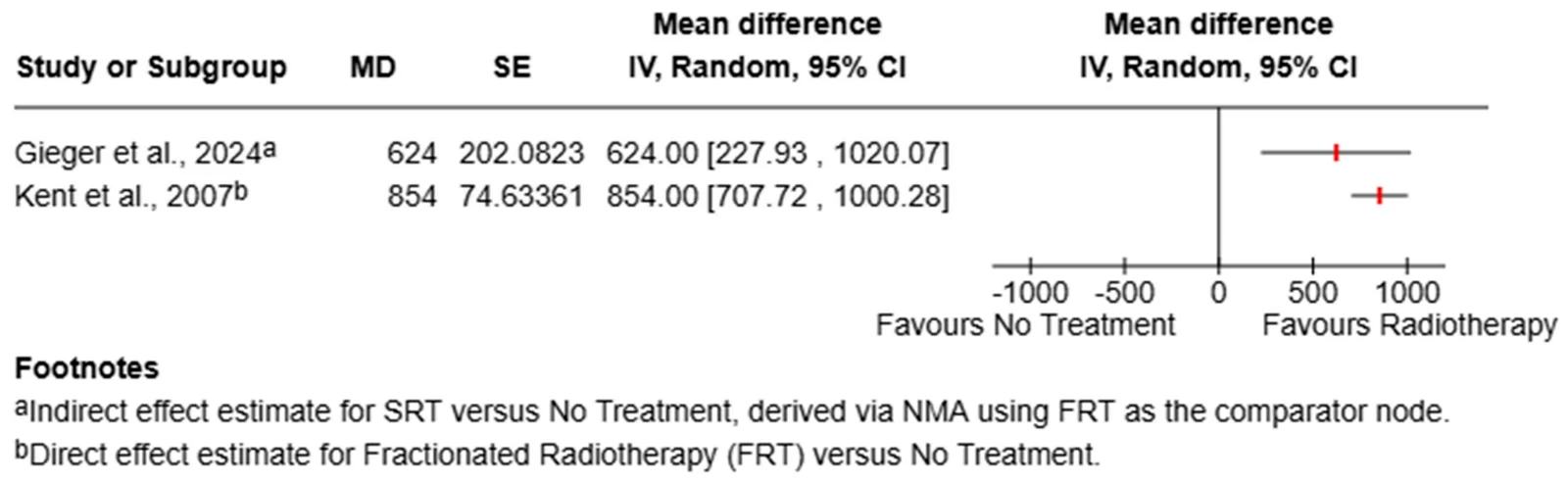
Forest plot comparing overall survival time (days) in dogs treated with FRT and SRT versus NT across included studies [21,22].

The network meta-analysis demonstrated statistically significant differences in clinical outcomes. Neurological outcomes are presented in Figure 8. Both radiotherapeutic interventions demonstrated statistically significant improvements compared to NT (SRT: OR = 109.17, 95% CI 3.99–2985.49; *p* = 0.0054; FRT: OR = 31.84, 95% CI 1.61–631.65; *p* = 0.0232). Survival time for the procedures is presented in Figure 9. Both radiotherapeutic interventions were associated with a statistically significant increase in survival time (SRT: MD = 624.00 days, 95% CI 227.93–1020.07; *p* = 0.002; FRT: MD = 854.00 days, 95% CI 707.72–1000.28; *p* < 0.0001).

## 4. Discussion

CS is a common endocrine disorder in dogs characterized by chronic exposure to excessive amounts of glucocorticoids, often affecting middle-aged to older animals [2,12]. It is primarily classified into PDH and ADH subtypes of the disorder. PDH accounts for most cases and results from excessive ACTH secretion associated with pituitary adenomas, whereas ADH is caused by cortisol-secreting adrenal tumors [2,3,4,5,26,27]. Clinical signs typically include polyuria, polydipsia, polyphagia, abdominal distension, and dermatologic abnormalities [5,6].

Although medical treatments remain the most used management strategy for CS in dogs, non-medical treatments, including surgical and radiotherapeutic interventions, can play an important role in select cases [15]. Non-medical interventions may offer better CS control. It can directly target the underlying cause of the disease (tumor), particularly when a cure is possible (tumor excision) or medical management is ineffective [16,17,18]. The findings of this study demonstrate that surgical and radiotherapeutic interventions provide different clinical benefits, and this depends on the underlying disease process, treatment protocol, and clinical application.

Surgical interventions include adrenalectomy and hypophysectomy; both of which directly remove the source of excessive hormone production. Adrenalectomy is the primary surgical treatment for dogs with ADH and involves excision of the affected adrenal gland [28]. This procedure can be performed using either OA or LA. OA is the surgical approach that is often selected for larger, locally invasive, or technically challenging adrenal tumors. Transabdominal and retroperitoneal approaches are the most commonly used techniques for OA, providing excellent visualization of the adrenal gland and facilitating surgical manipulation of the adrenal gland and surrounding tissues [29,30]. These approaches, however, require larger incisions and are associated with greater tissue trauma, increased risk of hemorrhage, wound dehiscence, and postoperative infection [31]. In contrast, LA is a minimally invasive, camera-assisted procedure that uses small incisions and is generally associated with reduced surgical trauma, shorter postoperative recovery, and improved perioperative outcomes [32,33,34].

The results demonstrated no statistically significant differences in survival between LA and OA at 12, 24, or 36 months [23,24,25]. This suggests that the available evidence does not support a difference in long-term survival between the two surgical approaches. Instead, long-term survival is likely influenced more by tumor specific factors, including tumor type, biological behavior, local invasion of surrounding tissues, metastatic disease, and the successful surgical excision of the mass [28]. In the study, LA was associated with shorter surgical times and reduced hospitalization compared with OA [23,24,25]. These perioperative benefits are likely attributable to the minimally invasive nature of LA, including reduced tissue trauma, smaller incisions, and reduced postoperative pain, and this likely assisted in the with faster recovery [24,25,32].

Similarly in human medicine, LA has been associated with improved perioperative outcomes, including lower morbidity, reduced blood loss, shorter hospitalization, and faster postoperative recovery compared with OA [33,34]. Additionally, long-term survival times in people have been reported to be comparable between LA and OA when tumor resection is successful [35]. This evidence aligns well with our current findings, and suggests that long-term survival is influenced more by tumor characteristics and the removal of the tumor than by the surgical approach. Consequently, the information indicates that the primary advantage of LA relates to improved perioperative recovery, with no additional long-term survival benefit when compared with OA. It should also be noted that LA requires advanced surgical training and specialized equipment, which may limit its availability in some clinical settings [32]. That said, when appropriate surgical expertise is available and tumor characteristics are suitable, LA may be considered the preferred surgical technique (improved perioperative outcomes) for adrenal gland tumors.

Radiotherapy represents a different type of treatment and is primarily used in cases of PDH, particularly when pituitary macroadenomas are present [36]. Radiotherapy, unlike surgery, does not excise the tumor but instead delivers targeted high energy ionizing radiation to reduce tumor size and control tumor growth. FRT delivers smaller doses of radiation over multiple sessions, which allows for a gradual reduction in tumor size. Conversely, SRT delivers a highly focused, high dose of radiation in fewer sessions, and this allows for more precise targeting of the tumor [35,37]. Radiotherapies are particularly important in cases where neurological signs are present, as they can reduce tumor-induced pressure (mass effect) on surrounding brain tissue and improve the clinical symptoms of the disease [37].

The results demonstrated time-dependent effects of radiotherapy on survival rates. At 12 months, only FRT demonstrated significantly improved survival compared with NT, whereas both FRT and SRT demonstrated significant survival benefits at 24 months. However, these survival advantages were not maintained at 36 months for either radiotherapeutic intervention [21,22]. This suggests that radiotherapy may provide effective short- to medium-term tumor control but may not prevent long-term tumor progression. The loss of a significant survival benefit at later time points may reflect continued tumor growth, recurrence, or progression of the disorder [34,35,36]. Moreover, radiotherapy was associated with improved neurologic outcomes as well as prolonged survival time. Reported neurologic improvements included reductions in seizures, obtundation, behavioral abnormalities, circling, appetite loss, and visual deficits associated with pituitary mass effects [21,22]. These findings suggest that radiotherapy may be particularly beneficial for PDH in dogs, especially in dogs presenting neurological abnormalities.

Interestingly, FRT and SRT are well-established treatments in human medicine, demonstrating effective local tumor control, reduced tumor progression, and improvements in neurological symptoms associated with pituitary mass effects [38]. Although tumor control can be achieved in many people, tumor progression and treatment-related complications are documented in long-term follow-up [39]. The information suggests that radiotherapy can be effective in controlling tumor growth and alleviating neurological signs but may not completely prevent long-term progression of the disease.

As described, the observed improvements in neurological outcomes and survival time are likely attributable to reduced tumor burden and thereby reducing the progression of PDH in dogs. However, these findings should be interpreted with caution, as data in the meta-analysis was likely influenced by differences in tumor size, tumor invasiveness, disease progression, and variations in treatment protocols. Furthermore, the available evidence was limited to only two retrospective studies with relatively small sample sizes and wide confidence intervals. This small sample size can reduce the precision and certainty of the estimated treatment effects. Consequently, larger prospective studies comparing FRT and SRT are needed to better define their long-term effectiveness and identify patient populations most likely to benefit from treatment. A final consideration—like all interventions, radiotherapies for the treatment of pituitary tumors are not without inherent risk. Morbidities related to treatment include injury to surrounding tissues, pituitary hemorrhage, visual and auditory impairments, long-term electrolyte disturbances, and other neurological deficits [40,41]. As such, clinicians should be aware of the potential treatment complications associated with radiotherapies in dogs with PDH.

Collectively, the findings in this meta-analysis study demonstrate differences in the effects of non-medical treatments. Surgical intervention directly removes the source of uncontrolled cortisol production and therefore has the potential to cure the disease in appropriately selected ADH dogs [23,24,25]. In contrast, radiotherapy primarily slows pituitary tumor growth and is associated with improved short- to medium-term survival and neurological outcomes in PDH dogs [21,22]. In general, these findings suggest that treatment selection should be guided by various criteria. These include disease subtypes, tumor characteristics, and factors such as surgical expertise and treatment availability. Adrenalectomy may be the preferred treatment for appropriately selected dogs with ADH, whereas radiotherapy may provide the greatest benefit for dogs with PDH, particularly those with pituitary macroadenomas and neurological abnormalities [21,22,23,24,25].

Several limitations should be considered when interpreting these findings. First, all included studies were retrospective cohort studies, making them susceptible to confounding and selection bias [20]. Factors such as disease severity, tumor size, tumor invasiveness, neurological status, clinician preference, patient comorbidities, and suitability for intervention may have influenced treatment selection and, consequently, treatment outcomes. Secondly, clinical heterogeneity existed across the included studies. More specifically, although adrenalectomy and radiotherapy were evaluated separately according to disease subtypes, differences remain in tumor characteristics, disease severity, treatment protocols, patient selection, clinician expertise, and follow-up duration. Furthermore, PDH and ADH can differ substantially in tumor biology, therapeutic indications, and prognosis. Collectively, these factors likely contributed to inter-study variability and influenced the pooled treatment estimates. Thirdly, the relatively small number of included studies and limited sample sizes reduced the precision of the pooled estimates, contributing to wide confidence intervals across several analyses. Finally, methodological heterogeneity further limited the strength of the available evidence. Differences in study design, outcome definitions, measurement methods, and follow-up assessments reduced the comparability of the included studies. As such, these limitations highlight the need for larger, well-designed prospective studies using standardized treatment protocols and outcome measures. This would better determine the long-term effectiveness of non-medical interventions for CS in dogs.

Despite these limitations, this study provides insights into the role of surgical and radiotherapeutic interventions with CS in dogs. Laparoscopic adrenalectomy offered clear perioperative advantages, including shorter surgical duration and reduced hospitalization times without compromising long-term survival rates. These findings suggest that LA may provide a less invasive alternative to OA while maintaining comparable survival rates. Moreover, radiotherapeutic intervention, FRT, demonstrated meaningful short-term survival benefits at 12 months, while both FRT and SRT demonstrated medium-term survival benefits at 24 months. Radiotherapy was also associated with improvements in neurologic outcomes, including reductions in seizures, obtundation, behavioral abnormalities, circling, appetite loss, and visual deficits associated with pituitary mass effects. These neurologic improvements are clinically important, as pituitary tumor progression can substantially reduce quality of life in affected dogs [21,22]. In general, the findings of this study support surgical and radiotherapeutic interventions for the treatment of dogs with naturally occurring CS in appropriately selected cases. To further advance the knowledge of non-medical treatment of dogs with CS, prospective studies with larger sample sizes, standardized treatment protocols, and longer follow-up periods to better evaluate long-term outcomes should be considered.

## 5. Conclusions

This meta-analysis and systematic review evaluated the application of non-medical treatments for CS in dogs. For ADH in dogs, LA was associated with shorter surgical times and hospitalization as compared with OA, while long-term survival outcomes were comparable between the surgical interventions. For PDH in dogs, radiotherapy was associated with improved neurologic outcomes, prolonged survival time, and improved short- to medium-term survival. These benefits in PDH dogs, however, were not maintained at longer intervals. The findings in this study suggest that non-medical interventions may provide clinical benefits when applied to the appropriate clinical cases of dogs with CS. The available information presented in this study, however, was limited by the small number of retrospective studies and as such should be interpreted carefully. Therefore, to improve the utility of this meta-analysis study, the addition of prospective studies with standardized outcome reporting should be considered. This would further enhance our understanding of effective non-medical treatment in dogs with CS and aid in implementing these therapies in veterinary medicine.

## Figures and Tables

**Table 1:** Key characteristics of included studies and the data extracted for analysis.

Author	Period	Study	Type	Sample Size	Age	Breed	Gender	Comparator	Outcome
Gieger et al., 2024 [21]	2016–2022	Retrospective	PDH	44 dogs (27 SRT, 17 FRT)	Median 10 years (SRT); 8 years (FRT)	Multiple breeds	20 M/22 F/2 I	SRT vs. FRT	MST 608 days; no survival difference between treatments; younger dogs had longer survival.
Kent et al., 2007 [22]	1998–2004	Retrospective	PDH	46 dogs (19 RT treated, 27 untreated)	Median 10.4 years (treated group); 11.3 years (control group)	Multiple breeds	NR	RT vs. no RT	RT significantly improved survival and neurologic outcomes.
Mayhew et al., 2014 [23]	2003–2013	Retrospective	ADH and mixed/unspecified	48 dogs (23 LA, 25 OA)	Median 11 years (laparoscopic); 10 years (open)	Multiple breeds	18 M/29 F/1 I	LA vs. OA	LA had shorter surgical and hospitalization times, with no perioperative deaths in the group; one LA dog excluded due to OA conversion; seven OA dogs excluded for missing surgical time records.
Taylor & Monnet, 2021 [24]	1995–2020	Retrospective	ADH and mixed/unspecified	40 dogs (14 LA, 26 OA)	Mean 10.6 years (LA); 10.8 years (OA)	Multiple breeds	14 M/25 F	LA vs. OA	LA had shorter surgical time and lower hypotension; survival did not differ between group.
van Bokhorst et al., 2023 [25]	2008–2022	Retrospective	ADH and mixed/unspecified	70 dogs (42 LA, 28 OA)	Mean 9.9 yrs (LA); 9.6 yrs (OA)	Multiple breeds	37 M/33 F	LA vs. OA	LA had shorter surgery and hospitalization time with lower complication rate; survival did not differ between group; One LA dog excluded due to unrelated catheter-site infection.

ADH, adrenal-dependent hypercortisolism; F, female; FRT, fractionated radiation therapy; I, intact; LA, laparoscopic adrenalectomy; M, male; MST, median survival time; NR, not reported; OA, open adrenalectomy; PDH, pituitary-dependent hypercortisolism; RT, radiation therapy; SRT, stereotactic radiation therapy.

## Data Availability

The original contributions presented in this study are included in the article/Supplementary Material. Further inquiries can be directed to the corresponding author.

## Supplementary Materials

The following supporting information can be downloaded at https://www.mdpi.com/article/10.3390/vetsci13080771/s1. Tables S1–S5 presents the search strategies for each database, and Tables S6–S10 presents the ROBINS-I risk of bias assessments of reach included article. Table S1. Ovid MEDLINE(R) (1946 to 3 October 2025). Table S2. Embase (1974 to 3 October 2025). Table S3. Web of Science (1955 to 3 October 2025). Table S4. Academic Search Complete (1998 to 3 October 2025). Table S5. Cochrane Library (to 3 October 2025). Table S6. Risk of bias assessment for Van Bokhorst, 2023. Table S7. Risk of bias assessment for Taylor & Monnet, 2021. Table S8. Risk of bias assessment for Mayhew et al., 2014. Table S9. Risk of bias assessment for Geiger et al., 2024. Table S10. Risk of bias assessment for Kent et al., 2007.

## Abbreviations

ADH	Adrenal-dependent hypercortisolism
ACTH	Adrenocorticotropic hormone
CI	Confidence interval
CS	Cushing’s syndrome
F	Female
FRT	Fractionated radiotherapy
HPA	Hypothalamic–pituitary–adrenal
I	Intact
LA	Laparoscopic adrenalectomy
M	Male
MD	Mean difference
MST	Median survival time
NT	No treatment
OA	Open adrenalectomy
OR	Odds ratio
PDH	Pituitary-dependent hypercortisolism
PRISMA	Preferred Reporting Items for Systematic Reviews and Meta-Analyses
RCT	Randomized controlled trial
ROBINS-I	Risk Of Bias In Non-randomized Studies of Interventions
RT	Radiotherapy
SD	Standard deviation
SEM	Standard error of the mean
SRT	Stereotactic radiotherapy
