# Supplementary material for: Effectiveness of Non-Medical Treatment on Survival, Perioperative, and Clinical Outcomes in Canine Cushing’s Syndrome: A Systematic Review and Network Meta-Analysis
Source: Vet Sci. 2026 Aug 1;13(8):771. doi: 10.3390/vetsci13080771 (PMC13517618; doi:10.3390/vetsci13080771)
Supplement: Supplementary file 1 [file vetsci-13-00771-s001.zip › Table_S1-S5_Search_Strategies.pdf]

Table S1. Ovid MEDLINE(R) (1946 to October 03, 2025).

#	Search Statement	Results
1	exp Dogs/	353031
2	(canine* or dog or dogs or doggy or puppy or puppies or mongrel* or hound or hounds or pooch* or mutt or mutts or bitch*).mp.	417637
3	(terrier* or spaniel* or retriever* or mastiff* or pinscher* or collie* or poodle* or dachshund* or corgi* or shepherd* or sheepdog* or beagle* or coonhound* or bloodhound* or borzoi* or english foxhound* or greyhound* or harrier* or irish wolfhound* or otterhound* or rhodesian ridgeback* or scottish deerhound*).mp. [mp=title, book title, abstract, original title, name of substance word, subject heading word, floating sub-heading word, keyword heading word, organism supplementary concept word, protocol supplementary concept word, rare disease supplementary concept word, unique identifier, synonyms, population supplementary concept word, anatomy supplementary concept word]	31219
4	1 or 2 or 3	423097
5	exp Cushing Syndrome/ or exp Pituitary ACTH Hypersecretion/ or exp ACTH-Secreting Pituitary Adenoma/ or exp Adrenal Gland Neoplasms/ or exp Adrenocorticotropic Hormone/ or exp Adrenocortical Hyperfunction/ or exp Hydrocortisone/	165243
6	(cushing* or adrenocorticotropic hormone* or ACTH* or ((corticotrop* or pituitary or adrenal) adj2 (adenoma* or tumo?r*)) or hyperadrenocortic* or hypercortisol* or hydrocortisone*).mp.	179828
7	5 or 6	210943
8	exp General Surgery/ or Hypophysectomy/ or exp Adrenalectomy/ or exp Radiotherapy/	292879
9	(surgical* or surger* or transspphenoidal* or hypophysectom* or adrenalectom* or neurosurger* or neuosurgical* or radiotherap* or radiotherapeutic* or radiosurger* or radiat* or cyberknife* or gamma knife* or resection* or excision*).mp.	4875086
10	8 or 9	4885960
11	4 and 7 and 10	822

Table S2. Embase (1974 to October 03, 2025).

#	Search Statement	Results
1	exp dogs/	305375
2	(canine* or dog or dogs or doggy or puppy or puppies or mongrel* or hound or hounds or pooch* or mutt or mutts or bitch*).mp.	392297
3	(terrier* or spaniel* or retriever* or mastiff* or pinscher* or collie* or poodle* or dachshund* or corgi* or shepherd* or sheepdog* or beagle* or coonhound* or bloodhound* or borzoi* or english foxhound* or greyhound* or harrier* or irish wolfhound* or otterhound* or rhodesian ridgeback* or scottish deerhound*).mp. [mp=title, abstract, heading word, drug trade name, original title, device manufacturer, drug manufacturer, device trade name, keyword heading word, floating subheading word, candidate term word]	43319
4	1 or 2 or 3	403830
5	exp cushing syndrome/ or exp cushing's disease/ or exp ACTH secreting adenoma/ or exp adrenal tumor/ or exp corticotropin/ or exp adrenal cortex hyperfunction/ or exp hypercortisolism/	133598
6	(cushing* or adrenocorticotropic hormone* or ACTH* or ((corticotrop* or pituitary or adrenal) adj2 (adenoma* or tumo?r*)) or hyperadrenocortic* or hypercortisol* or hydrocortisone*).mp.	272058
7	5 or 6	312425
8	exp general surgery/ or exp hypophysectomy/ or exp adrenalectomy/ or exp radiotherapy/	819387
9	(surgical* or surger* or transspphenoidal* or hypophysectom* or adrenalectom* or neurosurger* or neuosurgical* or radiotherap* or radiotherapeutic* or radiosurger* or radiat* or cyberknife* or gamma knife* or resection* or excision*).mp.	6808391
10	8 or 9	6836806
11	4 and 7 and 10	916

Table S3. Web of Science (1955 to October 03, 2025).

#	Search Statement	Results
1	canine* or dog or dogs or doggy or puppy or puppies or mongrel* or hound or hounds or pooch* or mutt or mutts or bitch* (Topic)	1,101,262
2	terrier* or spaniel* or retriever* or mastiff* or pinscher* or collie* or poodle* or dachshund* or corgi* or shepherd* or sheepdog* or beagle* or coonhound* or bloodhound* or borzoi* or “english foxhound*” or greyhound* or harrier* or “irish wolfhound*” or otterhound* or “rhodesian ridgeback*” or “scottish deerhound*” (Topic)	117,807
3	#1 OR #2	1,095,446
4	(cushing* or “adrenocorticotropic hormone*” or ACTH* or ((corticotrop* or pituitary or adrenal) NEAR/2 (adenoma* or tumo?r*)) or hyperadrenocortic* or hypercortisol* or hydrocortisone*) (Topic)	282,505
5	(surgical* or surger* or transspphenoidal* or hypophysectom* or adrenalectom* or neurosurger* or neuosurgical* or radiotherap* or radiotherapeutic* or radiosurger* or radiat* or cyberknife* or “gamma knife*” or resection* or excision*) (Topic)	14,939,401
8	#5 AND #3 AND #4 MEDLINE(R) (Exclude – Database)	706

Table S4. Academic Search Complete (1998 to October 03, 2025).

#	Search Statement	Results
1	canine* or dog or dogs or doggy or puppy or puppies or mongrel* or hound or hounds or pooch* or mutt or mutts or bitch*	171,705
2	terrier* or spaniel* or retriever* or mastiff* or pinscher* or collie* or poodle* or dachshund* or corgi* or shepherd* or sheepdog* or beagle* or coonhound* or bloodhound* or borzoi* or "english foxhound*" or greyhound* or harrier* or "irish wolfhound*" or otterhound* or "rhodesian ridgeback*" or "scottish deerhound*"	58,429
3	S1 OR S 2	215,054
4	(cushing* or "adrenocorticotropic hormone*" or ACTH* or ((corticotrop* or pituitary or adrenal) N2 (adenoma* or tumo?r*)) or hyperadrenocortic* or hypercortisol* or hydrocortisone*)	44,976
5	(surgical* or surger* or transsppphenoidal* or hypophysectom* or adrenalectom* or neurosurger* or neuosurgical* or radiotherap* or radiotherapeutic* or radiosurger* or radiat* or cyberknife* or "gamma knife*" or resection* or excision*)	3,002,352
8	S3 AND S4 AND S5	207

Table S5. Cochrane Library (to October 03, 2025).

#	Search Statement	Results
1	MeSH descriptor: [Dogs] explode all trees	728
2	canine* or dog or dogs or doggy or puppy or puppies or mongrel* or hound or hounds or pooch* or mutt or mutts or bitch*	4721
3	terrier* or spaniel* or retriever* or mastiff* or pinscher* or collie* or poodle* or dachshund* or corgi* or shepherd* or sheepdog* or beagle* or coonhound* or bloodhound* or borzoi* or (english NEXT foxhound*) or greyhound* or harrier* or (irish NEXT wolfhound*) or otterhound* or (rhodesian NEXT ridgeback*) or (scottish NEXT deerhound*)	3432
4	{or #1-#3}	8047
5	MeSH descriptor: [Cushing Syndrome] explode all trees	133
6	MeSH descriptor: [Pituitary ACTH Hypersecretion] explode all trees	49
7	MeSH descriptor: [ACTH-Secreting Pituitary Adenoma] explode all trees	28
8	MeSH descriptor: [Adrenal Gland Neoplasms] explode all trees	181
9	MeSH descriptor: [Adrenocorticotropic Hormone] explode all trees	1730
10	MeSH descriptor: [Adrenocortical Hyperfunction] explode all trees	294
11	MeSH descriptor: [Hydrocortisone] explode all trees	7692
12	(cushing* or (adrenocorticotropic NEXT hormone*) or ACTH* or ((corticotrop* or pituitary or adrenal) NEAR/2 (adenoma* or tumor* or tumour*)) or hyperadrenocortic* or hypercortisol* or hydrocortisone*)	14091
13	{or #5-#12}	14404
14	MeSH descriptor: [General Surgery] explode all trees	519
15	MeSH descriptor: [Hypophysectomy] explode all trees	30
16	MeSH descriptor: [Adrenalectomy] explode all trees	113
17	MeSH descriptor: [Radiotherapy] explode all trees	10086
18	(surgical* or surger* or transspphenoidal* or hypophysectom* or adrenalectom* or neurosurger* or neuosurgical* or radiotherap* or radiotherapeutic* or radiosurger* or radiat* or cyberknife* or (gamma NEXT knife*) or resection* or excision*)	447364
19	{or #14-#18}	447668
20	#4 AND #13 AND #19	27
