# Supplementary material for: Effectiveness of Non-Medical Treatment on Survival, Perioperative, and Clinical Outcomes in Canine Cushing’s Syndrome: A Systematic Review and Network Meta-Analysis
Source: Vet Sci. 2026 Aug 1;13(8):771. doi: 10.3390/vetsci13080771 (PMC13517618; doi:10.3390/vetsci13080771)
Supplement: Supplementary file 1 [file vetsci-13-00771-s001.zip › Table_S6-S10_Risk_of_Bias_Assessments.pdf]

Table S6. Risk of bias assessment for Van Bokhorst, 2023.

Bias Domain	Assessment Justification	Risk of Bias Judgement
Bias due to confounding	Treatment selection (LA vs OA) based on tumor characteristics and surgeon choice; no adjustment performed.	Serious
Bias in classification of interventions	Clear distinction between laparoscopic and open procedures.	Low
Bias in selection of participants into the study	Surgical candidates reflect clinician selection; may exclude high-risk dogs.	Low
Bias due to deviations from intended interventions	Few deviations; conversions reported.	Low
Bias due to missing data	Near-complete follow-up.	Low
Bias in measurement of outcomes	Assessors unblinded; recurrence measurement variable.	Moderate
Bias in selection of the reported results	No preregistered protocol; selective analysis possible.	Moderate
Overall risk of bias	Driven by non-random allocation and unadjusted confounding.	Serious

Table S7. Risk of bias assessment for Taylor & Monnet, 2021.

Bias Domain	Assessment Justification	Risk of Bias Judgement
Bias due to confounding	Intervention selected by surgeon based on tumor characteristics; no adjustment.	Serious
Bias in classification of interventions	LA and OA are clearly defined.	Low
Bias in selection of participants into the study	Excluded unstable non-surgical dogs; cohort not representative	Moderate
Bias due to deviations from intended interventions	Standard treatment; minor risk from surgeon learning curve.	Low
Bias due to missing data	Outcomes nearly complete.	Low
Bias in measurement of outcomes	Subjective intraoperative complications; no blinding.	Moderate
Bias in selection of the reported results	No preregistration; limited outcome analysis.	Moderate
Overall risk of bias	Confounding dominates the risk profile.	Serious

Table S8. Risk of bias assessment for Mayhew et al., 2014.

Bias Domain	Assessment Justification	Risk of Bias Judgement
Bias due to confounding	No adjustment; intervention chosen based on tumor size and invasiveness.	Moderate
Bias in classification of interventions	LA and OA are clearly defined.	Low
Bias in selection of participants into the study	Only surgical candidates included; selection based on clinician judgment.	Moderate
Bias due to deviations from intended interventions	Minimal deviations; consistent perioperative care.	Low
Bias due to missing data	Outcomes nearly complete.	Low
Bias in measurement of outcomes	Unblinded assessors; some subjective complications.	Moderate
Bias in selection of the reported results	No preregistration; selective reporting possible.	Moderate
Overall risk of bias	Driven by confounding and retrospective study design.	Serious

Table S9. Risk of bias assessment for Geiger et al., 2024.

Bias Domain	Assessment Justification	Risk of Bias Judgement
Bias due to confounding	Treatment allocation based on clinical/tumor factors; no adjustment.	Serious
Bias in classification of interventions	SRT and FRT are clearly documented.	Low
Bias in selection of participants into the study	Only dogs completing RT included; prognosis skew	Serious
Bias due to deviations from intended interventions	Protocols followed; minimal deviations.	Low
Bias due to missing data	Some incomplete imaging follow-up; early deaths.	Moderate
Bias in measurement of outcomes	Unblinded assessors; subjective neurologic outcomes.	Moderate
Bias in selection of the reported results	No protocol; selective detail depth.	Moderate
Overall risk of bias	Confounding dominates risk profile	Serious

Table S10. Risk of bias assessment for Kent et al., 2007.

Bias Domain	Assessment Justification	Risk of Bias Judgement
Bias due to confounding	RT vs untreated allocation highly confounded by prognosis; no adjustment.	Serious
Bias in classification of interventions	Clear treated vs untreated groups.	Low
Bias in selection of participants into the study	Treated and untreated groups differ fundamentally at baseline.	Serious
Bias due to deviations from intended interventions	Untreated dogs received inconsistent care; RT protocol uniform.	Moderate
Bias due to missing data	Follow-up is more complete in the RT group.	Serious
Bias in measurement of outcomes	Unblinded assessors; subjective neurologic outcomes.	Moderate
Bias in selection of the reported results	No protocol; selective reporting possible.	Moderate
Overall risk of bias	Confounding and selection bias undermine internal validity.	Serious
